# Arginase II Promotes Intervertebral Disc Degeneration Through Exacerbating Senescence and Apoptosis Caused by Oxidative Stress and Inflammation *via* the NF-κB Pathway

**DOI:** 10.3389/fcell.2021.737809

**Published:** 2021-12-03

**Authors:** Fudong Li, Xiaofei Sun, Bing Zheng, Kaiqiang Sun, Jian Zhu, Chenglong Ji, Feng Lin, Le Huan, Xi Luo, Chen Yan, Jiashun Xu, Yun Hong, Yuan Wang, Ximing Xu, Jingchuan Sun, Zheming Song, Fanqi Kong, Jiangang Shi

**Affiliations:** ^1^Department of Orthopedic Surgery, Spine Center, Changzheng Hospital, Naval Medical University, Shanghai, China; ^2^The 905th Hospital of the People’s Liberation Army Navy of China, Shanghai, China

**Keywords:** ARG2, intervertebral disc degeneration, nucleus pulposus, oxidative stress, inflammatory response, senescence, apoptosis

## Abstract

Intervertebral disc degeneration (IDD) has been generally accepted as the major cause of low back pain (LBP), which imposes massive clinical and socioeconomic burdens. Previous studies have demonstrated that oxidative stress and inflammation-induced senescence and apoptosis of nucleus pulposus cells (NPCs) are the main cellular processes that cause IDD. Arginase II (ARG2), an enzyme involved in a variety of pathological processes, including cellular senescence, apoptosis, oxidative stress, and inflammation, has been shown to promote degeneration in several degenerative diseases, including osteoarticular diseases. Based on previous studies, we hypothesized that ARG2 deficiency might be conducive to the treatment of IDD by inhibiting the dyshomeostasis of the extracellular matrix (ECM), and the oxidative stress and inflammatory response-induced senescence and apoptosis *via* NF-κB. In this study, we found that ARG2 deficiency inhibited senescence and apoptosis of NPCs, and degeneration of the ECM induced by oxidative stress and the inflammatory response. Similar results were found with the selective NF-κB pathway inhibitor JSH-23. In contrast, overexpression of ARG2 had the opposite effect. Taken together, our results suggest that ARG2 deficiency prevents IDD *via* NF-κB, and may therefore, be a potential therapeutic strategy for IDD.

## Introduction

Intervertebral disc degeneration (IDD), the major cause of low back pain (LBP), is prevalent around the world and is a significant socio-economic burden (Blanquer et al., 2015). IDD is associated with both genetic and lifestyle factors (Elfering et al., 2002; Feng et al., 2016). Elucidating the precise mechanisms of IDD would allow for the development of novel therapeutic strategies for LBP. Increased nucleus pulposus cell (NPC) senescence and programmed cell death induced by the inflammatory response contribute to the development of IDD. Oxidative stress has also been reported to be closely related to IDD pathogenesis (Feng et al., 2017). The imbalance between reactive oxygen species (ROS) production and antioxidant scavenging can damage lipids, proteins, and DNA (Tang et al., 2018), which subsequently lead to apoptosis of NPCs. Collectively, these mechanisms lead to the impairment of intervertebral disk function.

Arginase, an enzyme that catalyzes the hydrolysis of arginine to ornithine and urea, plays a vital role in the pathogenesis of many diseases associated with cellular senescence, apoptosis, oxidative stress, and inflammation, including metabolic diseases and osteoarticular diseases (Yepuri et al., 2012; Suwanpradid et al., 2014; Caldwell et al., 2015; Choi W. S. et al., 2019). Two forms of arginase, arginase I, and arginase II (ARG2), are localized in the cytoplasm and mitochondria, respectively. Arginase I is mainly expressed in the liver, while ARG2 is expressed primarily in the extrahepatic tissues. It has been reported that mice with an ARG2 gene deficiency have a significant lifespan extension compared to wild type mice (Xiong et al., 2017). Recently, ARG2 was shown to cause destruction of osteoarthritis cartilage by upregulating the level of matrix metalloproteinases (MMPs) in chondrocytes (Choi W. S. et al., 2019). Since articular cartilage and nucleus pulposus are composed of similar cell types (fibrochondrocytes, chondrocytes, and chondrocyte-like cells), and since these tissues assume similar functions (distribution and transfer of weight across surfaces) (Kushioka et al., 2020), it may be presumed that their regenerative approaches would also be similar. However, the precise role of ARG2 in IDD has not yet been reported.

NF-κB, an inducible transcription factor, is composed of five members of the Rel family, including NF-κB1 (p105/p50), NF-κB2 (p100/p52), RelA (p65), RelB, and c-Rel (Choi M. C. et al., 2019). Among the NF-κB dimers, the p65/p50 heterodimer is found in most cell types and acts as a potent transcription factor (Choi M. C. et al., 2019). NF−κB is widely activated in the tumor microenvironment, immune responses, inflammatory responses, and cellular differentiation, and plays essential roles in inflammation, oxidative stress, senescence, and apoptosis in IDD (Choi M. C. et al., 2019; Dong et al., 2019; Yi et al., 2019; Shao et al., 2020, 2021; Zhang et al., 2021). In addition, NF-κB is reportedly activated by enzymatic and non-enzymatic ARG2, which induces upregulation of MMP3 and MMP13 in chondrocytes (Choi W. S. et al., 2019). However, it remains unclear whether ARG2 modulates the NF-κB pathway in intervertebral disks.

In this study, we examined the expression of ARG2 in human nucleus pulposus tissues. We elucidated the effects of ARG2 on the extracellular matrix (ECM), senescence, apoptosis, oxidative stress, and the inflammatory response of NPCs. We further studied the reciprocity between ARG2 and the NF-κB pathway to investigate the potential mechanisms of NPC survival induced by ARG2. Our study indicated that ARG2 could be a potential therapeutic target for IDD and confirmed that ARG2 deficiency prevents IDD by inhibiting expression of ECM-degrading enzymes, and ameliorating senescence, apoptosis, oxidative stress, and the inflammatory response in NPCs.

## Materials and Methods

### Tissue Sample Collection

Written informed consent was obtained from patients and their relatives before obtaining the nucleus pulposus tissue during surgery. This study was approved by the Ethics Committee of Shanghai Changzheng Hospital (ID number: 2017SL040). The experiments in this study were carried out in biosafety laboratory according to the Laboratory Biosafety Manual in our laboratory. Human normal disk tissue was taken from surgical patients with or lumbar trauma (Pfirrmann grade I, *n* = 9, age 27–53 years, mean 42 years). Degenerated disk tissue was collected from patients with lumbar disk herniation (Pfirrmann grade II–III, *n* = 6, age 28–70 years, mean 51 years; and Pfirrmann grade IV–V, *n* = 6, age 38–75 years, mean 53 years). The degree of IDD was evaluated by the Pfirrmann grading system. Three individual samples in each group were used for immunohistochemistry staining. Tissue protein was extracted from three samples in each group. NPCs were isolated from nucleus pulposus tissue in three patients with congenital scoliosis or lumbar trauma but not with IDD, because IDD is characterized by the loss of NPCs and phenotypic abnormalities (Zheng et al., 2020).

### Isolation and Culture of Human Primary Nucleus Pulposus Cells and the Establishment of a Degeneration Model With Interleukin 1β

Nucleus pulposus cells were isolated from different donors and these cells were used for the cell-based *in vitro* experiments in this study. The isolation process of NPCs was performed as described previously (Zhang et al., 2018). Nucleus pulposus tissue samples were washed three times with PBS, then cut and digested using 0.25% trypsin (Servicebio, Wuhan, China) and 0.01% EDTA at 37°C for approximately 45 min. Next, tissue pieces were digested by type II collagenase (Servicebio) for 4 h at 37°C. The cell suspension was sieved through a cell strainer (70 μm; Beyotime, Shanghai, China) and centrifuged at 1,000 × *g* for 5 min. NPCs were resuspended in DMEM/F12 with 10% fetal bovine serum (FBS, Gibco) and 1% penicillin/streptomycin (Beyotime) and cultured in a humidified atmosphere of 5% CO_2_ at 37°C, changing medium every 3 days. Cells at second passage were used for subsequent experiments. To establish the degeneration model of NPCs, cells were incubated for 24 h then treated with Recombinant Human Interleukin 1β (IL-1β) (10 ng/mL, Beyotime) for 24 or 48 h. Total RNA or protein were then extracted (Bai et al., 2019; Zhang et al., 2019).

### Real-Time Quantitative Reverse Transcription PCR

Total RNA was extracted from NPCs using the HiPure Total RNA Mini Kit (Magen, China) according to the manufacturer’s instructions. The HiScript^®^ III RT SuperMix for qPCR Kit (R323-01, Vazyme, Nanjing, China) was used to conduct reverse transcription with a 20 μl final reaction mixture according to the manufacturer’s instructions. Real-time PCR was performed using ChamQ^TM^ Universal SYBR qPCR Master Mix on an ABI 7500 Real-Time PCR system (Applied Biosystems, Foster City, CA, United States). The relative fold changes in mRNA expression were calculated using the 2^–ΔΔCt^ method. The sequences of primers used in this study are shown in Table 1.

**TABLE 1:** The sequences of primers used for quantitative reverse transcription PCR (qRT-PCR).

**Gene**	**Forward (5′–3′)**	**Reverse (5′–3′)**
ARG2	GCATTTGACCCTACACTGG	TCTTCGCCTCTTCCTCTG
MMP3	TTTTCTCCTGCCTGTGCT	TTCACGCTCAAGTTCCCT
MMP13	ATCTGAACTGGGTCTTCCAA	GCCTGTATCCTCAAAGTGAAC
Aggrecan	TGCAGAACAGTGCCATCA	CTCCATAGCAGCCTTCCC
Type II collagen	GGATGGCTGCACGAAAC	CCCTATGTCCACACCGAAT

### Western Blotting

Total cellular protein was extracted using radioimmunoprecipitation assay (RIPA) lysis buffer (Beyotime) and quantified using the bicinchoninic acid (BCA) protein assay kit (Beyotime). Protein extraction was performed in a similar manner for both tissues and cells in culture. Equal quantities of total protein were separated by 10% sodium dodecyl sulphate-polyacrylamide gel electrophoresis (SDS-PAGE), and then transferred to polyvinylidene fluoride (PVDF) membranes (Millipore, Billerica, MA, United States) by electroblotting. The membranes were blocked using tris-buffered saline tween-20 (TBST) containing 5% skimmed milk at 25°C for 2 h, then incubated with primary antibody diluent of the target protein overnight at 4°C. Immunolabeling was detected using a Tanon Imaging System (version 5200, Tanon Science & Technology Co., Ltd., Shanghai, China). The following primary antibodies were used: ARG2 (CST, #55003), TNF-α (CST, #3707), IL-6 (CST, #3732), COX2 (CST, #12282), iNOS (CST, #20609), Iκ-Bα (CST, # 9247), p- Iκ-Bα (CST, # 2859), MMP3 (Zenbio, Chengdu, China, 380816), MMP13 (Zenbio, 820098), type II collagen (Abcam, ab34712), aggrecan (Abcam, ab36861), Bcl-2 (Zenbio, 381702), Bax (Zenbio, 200958), cleaved caspase3 (CST, #9664), P16INK4a (CST, #80772), and P21 (Abcam, ab109520). GAPDH (CST, #5174) was used as the protein loading control. The protein bands were quantified using ImageJ and normalized to GAPDH.

### Small Interfering RNA Transfection

We silenced the expression of ARG2 in NPCs using small interfering RNA (siRNA) technology. siRNAs were purchased from GenePharma Co., (Shanghai, China). The ARG2 siRNA sequences were: GCUGAGGAAAUACACAAUATT (sense 5′-3′) and UAUUGUGUAUUUCCUCAGCTT (antisense 5′-3′). Briefly, a total of 5 × 10^5^ cells/well was seeded in 12-well plates. After 24 h, cells were transfected with ARG2 siRNA or control siRNA duplexes according to the manufacturer’s instructions. After 4 h, the medium was replaced with complete medium and IL-1β was added to the medium at a final concentration of 50 ng/ml. After 24 h incubation, RNA was extracted.

### Plasmid Construction and Transfection

The plasmid containing full-length ARG2 and a negative control plasmid were designed by GenePharma (Shanghai, China). Packaging of viral vectors was performed as described previously (Kong et al., 2020), and a titers test was performed. NPCs were plated at a density of 3 × 10^5^ cells/well in 24-well plates. After 24 h, the cells were co-transfected with the recombinant plasmid using Lipofectamine 2000 transfection reagent. The medium was replaced 4 h later with fresh medium. The overexpression efficacy of ARG2 was verified by qPCR and western blotting.

### Immunohistochemistry

Nucleus pulposus tissues from patients with lumbar trauma and patients with moderate and severe IDD were collected and fixed in 4% paraformaldehyde. Immunohistochemical staining was performed to locate ARG2 in human nucleus pulposus tissues as described previously (Tang et al., 2019). Sections were incubated with 0.1% trypsin for antigen retrieval for 30 min at 37°C, followed by blocking with bovine serum albumin (BSA) at room temperature for 15 min. Then, sections were incubated with a primary antibody against ARG2 (CST, #55003, 1:1,000) at 4°C overnight, followed by the secondary antibody (Servicebio, GB23303) and counterstaining with hematoxylin. Images were visualized by light microscopy (Olympus, Japan). Quantification analysis was performed using ImageJ. Immunostaining was independently assessed by three histopathologists who were blinded to the data and outcomes of the patients.

### Immunofluorescence

For type II collagen and MMP13 immunofluorescence staining, NPCs were fixed in 4% paraformaldehyde for 15–20 min, then permeabilized using 0.1% v/v Triton X-100 for 5 min. Samples were incubated with primary antibodies against type II collagen (1:100 – 1:500, Solarbio, K009364P), MMP13 (1:400 – 1:1,600, Servicebio, GB11247), and NF-κB p65 (1:1,000, Cell Signaling Technology, Inc., Danvers, MA, United States, D14E12, #8242) diluted in 0.2% w/v BSA-TBS for 1 h. After washing with PBS, samples were incubated with DAPI solution (Servicebio, G1012) for visualization of the nuclei followed by incubation with FITC-conjugated goat anti-rabbit IgG (Servicebio, GB22303) and Cy3-conjugated goat anti-rabbit IgG (Servicebio, GB21301) for 30 min in the dark. Fluorescence detection was performed by fluorescence microscope (Olympus, Japan). Immunofluorescence quantification was performed using ImageJ, following the ImageJ User Guide.

### Mitochondrial Membrane Potential Measurement

JC-1 is a fluorescent probe commonly used to measure mitochondrial membrane potential (MIMP). NPCs were seeded in 6-well plates for 24 h. JC-1 (200X) was added to ultrapure water and JC-1 staining buffer (5X) to make the JC-1 working solution. The JC-1 working solution was added into the wells and incubated at 37°C for 20 min. Samples were then washed twice with JC-1 staining buffer (1X) and visualized by confocal microscopy (Carl Zeiss LSM 700). Quantification was performed using ImageJ, following the ImageJ User Guide.

### Apoptosis Analysis

Apoptosis was detected by the terminal deoxynucleotidyl transferase (TdT) dUTP nick end labeling (TUNEL) method. NPCs were cultured in six-well plates. After fixing in 4% paraformaldehyde for 1 h, samples were permeabilized with 0.3% Triton X-100 for 10 min. Samples were washed twice with PBS, then incubated with the TUNEL detection solution for 60 min at 37°C. Next, samples were washed three times with PBS, followed by incubation with DAPI solution. Finally, TUNEL-positive cells were detected by fluorescence microscopy (570 nm, Olympus, Japan). The number of TUNEL-positive cells was counted in three randomly selected fields. Quantification was performed using ImageJ, following the ImageJ User Guide.

### SA-β-Gal Staining

Cells were fixed with 0.2% glutaraldehyde at room temperature for 15 min. After washing twice with PBS, samples were incubated with SA-β-gal working solution at 37°C overnight. Images were captured with a light microscope (Olympus, Japan). Quantification was performed using ImageJ, following the ImageJ User Guide.

### Reactive Oxygen Species Assay

Reactive oxygen species was detected using a ROS Assay Kit (Beyotime). Briefly, cells were incubated with DCFH-DA (diluted to 1:1,000 in serum-free medium) at 37°C for 20 min, then washed three times in serum-free cell culture medium to remove excess fluorescent probes. Samples were visualized by confocal microscopy (Carl Zeiss LSM 700). Quantification was performed using ImageJ, following the ImageJ User Guide.

### ELISA Assay

The concentration of TNFα and IL-1β in cell culture supernatants was measured using an ELISA kit (Abcam, ab181421, ab214025) according to the manufacturer’s instructions.

### Statistical Analysis

Experiments were carried out more than three times. Quantification of immunohistochemical analyses, immunofluorescence, and western blotting data were performed using ImageJ software. All quantitative data are presented as the mean ± standard deviation. Two-group comparisons were analyzed by Student’s *t*-test, and three or more group comparisons were performed by one-way analysis of variance. Immunohistochemical data were analyzed by unpaired Students *t*-test. Non-parametric data (Pfirrmann grading) were analyzed by the Kruskal–Wallis H test. Statistical analyses were performed using SPSS version 18.0 (IBM, Armonk, NY, United States). ^∗^*P* < 0.05, ^∗∗^*P* < 0.01, and ^∗∗∗^*P* < 0.001 were considered to be statistically significant.

## Results

### Arginase 2 Is Highly Expressed in Degenerated Intervertebral Disk

**FIGURE 1:**
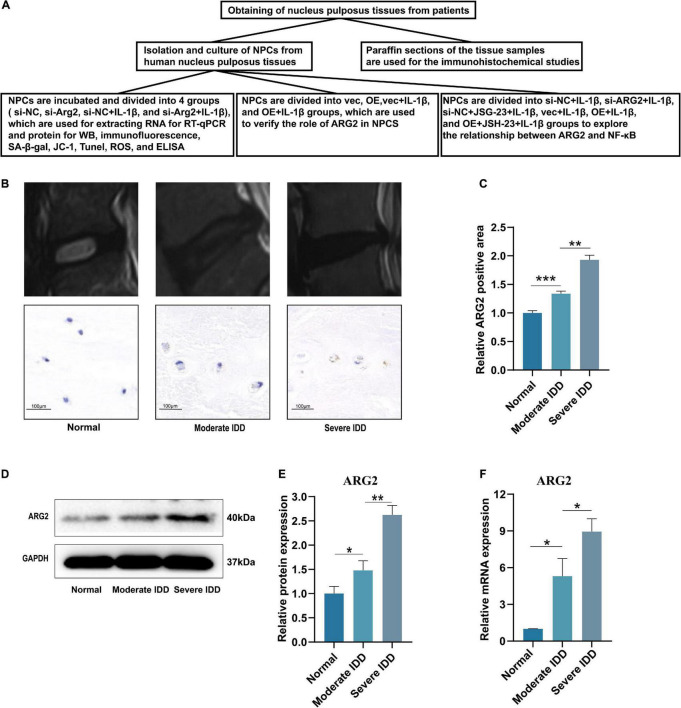
Arginase II (ARG2) expression is upregulated in intervertebral disc degeneration (IDD) tissues. **(A)** The flow diagram of this study. **(B)** Representative T2 signal MRI images and immunohistochemistry results (Scale bar: 100 μm) of each group. **(C)** Immunohistochemical results were analyzed by semiquantitative analysis. **(D)** The protein expression of ARG2 in nucleus pulposus tissues in each group was measured by western blotting. **(E)** The western blotting results were analyzed by semiquantitative analysis. **(F)** The mRNA level of ARG2 in the in nucleus pulposus tissues was measured. Data are represented as the mean ± SD. Two-group comparisons were analyzed by Student’s *t*-test. Significant differences between the treatment and control groups are indicated as *P < 0.05, **P < 0.01, and ***P < 0.001, *n* = 3.

The flow diagram of this study is shown in Figure 1A. The expression of ARG2 during the development of IDD was examined by immunohistochemical staining, quantitative reverse transcription PCR (qRT-PCR), and western blotting. Normal nucleus pulposus tissues were obtained from patients diagnosed with lumbar trauma and the degenerated nucleus pulposus tissues were acquired from patients with IDD. The intervertebral disk samples were divided into three groups [grade I: normal (the nucleus pulposus tissues were all from patients with lumbar trauma), *n* = 3; grade II–III: moderate degeneration, *n* = 3; and grade IV–V: severe degeneration, *n* = 3] according to the modified Pfirrmann grading system. Immunohistochemistry indicated that ARG2 expression was significantly higher in degenerated intervertebral disk than normal intervertebral disk. Furthermore, ARG2 expression was higher in severe IDD than moderate IDD (Figures 1B,C). The expression of ARG2 was also examined in nucleus pulposus tissues from patients with lumbar vertebral fracture or IDD. Compared with normal nucleus pulposus tissues, the mRNA and protein levels of ARG2 were significantly higher in both moderately and severely degenerative nucleus pulposus tissues (Figures 1D–F), suggesting that ARG2 was involved in the degeneration of human intervertebral disk.

### Interleukin 1β Promotes Arginase 2 Expression in Nucleus Pulposus Cells

**FIGURE 2:**
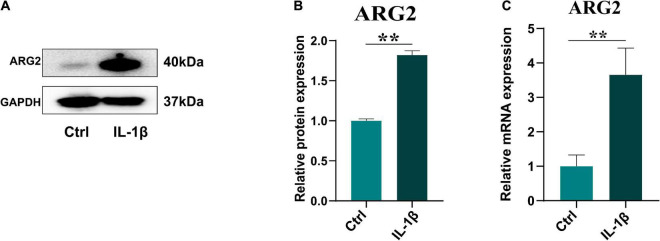
Interleukin 1β (IL-1β) promoted the expression of ARG2 in nucleus pulposus cells (NPCs). **(A)** The western blotting showed that the protein expression of ARG2 in NPCs was upregulated *via* the treatment of IL-1β. **(B)** The western blotting results were analyzed by semiquantitative analysis. **(C)** The mRNA level of ARG2 in NPCs was enhanced after the treatment of IL-1β. Data are represented as the mean ± SD. Two-group comparisons were analyzed by Student’s *t*-test. Significant differences between the treatment and control groups are indicated as *P < 0.05, **P < 0.01, and ***P < 0.001, *n* = 3.

The effects of IL-1β on ARG2 mRNA and protein expression were examined in NPCs that had been treated with IL-1β (10 ng/ml) for 24 h. ARG2 protein levels were significantly enhanced by IL-1β treatment (Figures 2A,B). Similarly, qRT-PCR analysis revealed that IL-1β promoted ARG2 mRNA expression (Figure 2C). Taken together, our results suggest that IL-1β could promote the expression of ARG2 in NPCs.

### The Effects of Arginase 2 Silencing on the Expression of Matrix Metalloproteinases, Type II Collagen, and Aggrecan in Nucleus Pulposus Cells

**FIGURE 3:**
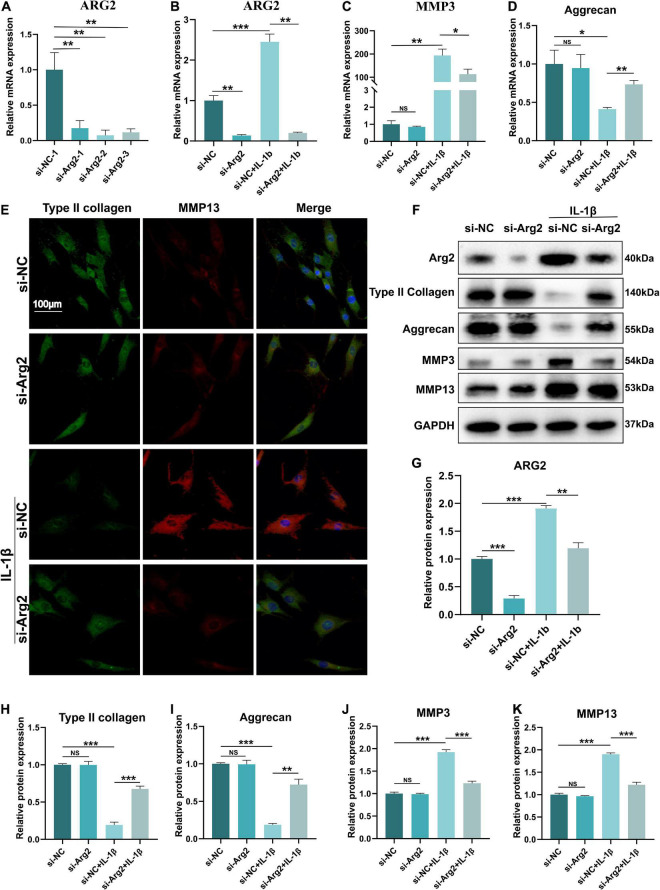
The effect of ARG2 silencing on the expression of matrix metalloproteinases (MMPs), type II collagen, and aggrecan in NPCs. **(A)** The silencing efficiency of ARG2 was selected. **(B)** The efficacy of small interfering RNA (siRNA)-ARG2 in NPCs treated with IL-1β. **(C,D)** The effect of ARG2 downregulation on the mRNA levels of MMP3 and aggrecan of NPCs treated with IL-1β. **(E)** MMP13 and type II collagen levels were detected by immunofluorescence (Scale bar: 100 μm). **(F–K)** The effect of ARG2 downregulation on the protein levels of ARG2, type II collagen, aggrecan, MMP3, and MMP13 of NPCs treated with IL-1β. Data are represented as the mean ± SD. Two-group comparisons were analyzed by Student’s *t*-test. Significant differences between the treatment and control groups are indicated as *P < 0.05, **P < 0.01, and ***P < 0.001, *n* = 3.

Three specific siRNAs for ARG2 and a control siRNA were used in this study, and the siRNA with the highest silencing efficiency was selected for subsequent experiments (Figure 3A). ARG2 mRNA levels were significantly reduced by siRNA treatment, demonstrating that ARG2 expression was successfully silenced in human NPCs. The efficacy of siRNA-ARG2 in NPCs treated with IL-1β was assessed by measuring ARG2 mRNA levels. We found that siRNA-ARG2 could also significantly reduce ARG2 levels under these conditions (Figure 3B). Degradation of the ECM is a primary factor contributing to the pathogenesis of IDD. To investigate the effect of ARG2 downregulation on ECM degradation, we examined MMP3 and aggrecan mRNA levels in NPCs transfected with siRNA−NC or siRNA−ARG2 and treated with IL-1β. We found that ARG2 silencing reduced MMP3 mRNA expression while enhancing aggrecan mRNA levels in the NPCs treated with IL-1β (Figures 3C,D). Immunofluorescence staining revealed that siRNA-ARG2 significantly reduced MMP13 expression and increased type II collagen levels in NPCs induced by IL-1β (Figure 3E). Moreover, the effects of ARG2 silencing on ARG2, type II collagen, aggrecan, MMP3, and MMP13 proteins expression were consistent with these findings. A significant increase in type II collagen and aggrecan protein expression, and significant reduction in MMP3 and MMP13 levels were observed in the ARG2 silencing NPCs treated with IL-1β (Figures 3F,H–K). Taken together, our data suggest that downregulation of ARG2 ameliorates NPC ECM degradation.

### Silencing of Arginase 2 Alleviates Interleukin 1β-Induced Senescence and Apoptosis in Nucleus Pulposus Cells

**FIGURE 4:**
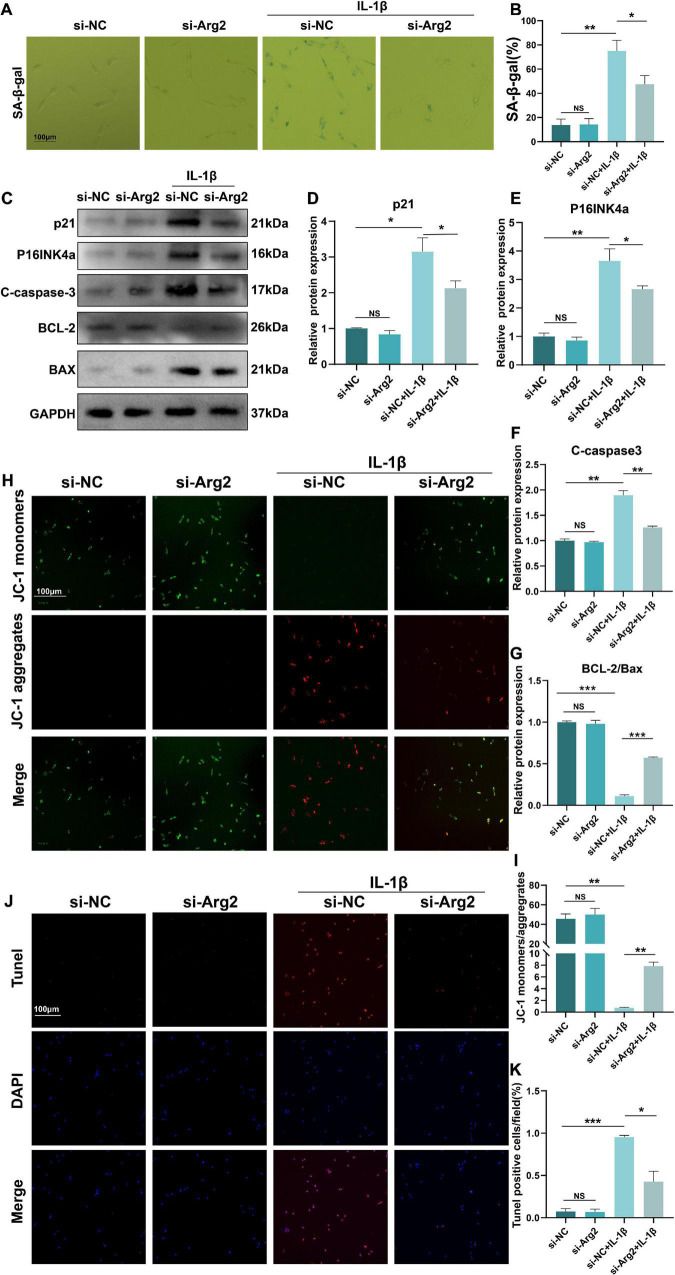
The silencing of ARG2 alleviated IL-1β-induced senescence and apoptosis in NP cells. **(A,B)** SA-β-gal staining and the ratio of SA-β-gal-positive cells. **(C–E)** The protein level of senescent marker p21 and P16INKa were detected *via* western blotting. **(C,F,G)** The protein level of apoptosis marker Cleaved-caspase-3, BCL-2, and BAX were detected *via* western blotting. **(H,I)** Mitochondrial membrane potential (MIMP) reflecting the early stage of apoptosis was assessed to detect apoptosis *via* JC1 staining (Scale bar: 100 μm). **(J,K)** The apoptosis rate was assessed with TUNEL staining (Scale bar: 100 μm). Data are represented as the mean ± SD. Two-group comparisons were analyzed by Student’s *t*-test. Significant differences between the treatment and control groups are indicated as *P < 0.05, **P < 0.01, and ***P < 0.001, *n* = 3.

The pathogenesis of IDD is closely associated with cellular senescence and apoptosis of NPCs. To quantify the number of senescent cells, we performed SA-β-gal staining and calculated the ratio of SA-β-gal-positive cells of the IL-1β-treated NPCs (Figures 4A,B). We found that siRNA-ARG2 significantly reduced the number of SA-β-gal-positive senescent NPCs. Consistent with these findings, we showed that protein expression levels of the senescence markers, P16INK4a and p21, were significantly reduced by siRNA-ARG2 (Figures 4C–E). Thus, our data indicated that silencing ARG2 alleviated IL-1β-induced cellular senescence in NPCs. Next, we examined the effects of ARG2 silencing on apoptosis. Western blot analysis revealed that siRNA-ARG2 decreased the levels of apoptotic cleaved-caspase 3 and Bax, while increasing expression of the anti-apoptotic Bcl2 compared with the IL-1β treatment group (Figures 4C,F,G). Changes in the MIMP reflect mitochondrial dysfunction during the early stages of apoptosis. Thus, we used JC-1 staining to detect MIMP and measure early apoptosis. Monomeric JC-1 (green fluorescent) represents high-MIMP, which reversibly transforms into aggregated JC-1 (red fluorescent) with loss of MIMP. Our data showed that siRNA-ARG2 significantly improved the MIMP inhibited by IL-1β (Figures 4H,I). Finally, we assessed the rate of apoptosis with TUNEL staining. We found that ARG2 silencing markedly reduced the number of TUNEL-positive cells induced by IL-1β (Figures 4J,K). Taken together, our data indicate that siRNA-ARG2 ameliorated cellular IL-1β-induced senescence and apoptosis in NPCs.

### The Effects of Arginase 2 Silencing on Oxidative Stress and the Inflammatory Response in Nucleus Pulposus Cells

**FIGURE 5:**
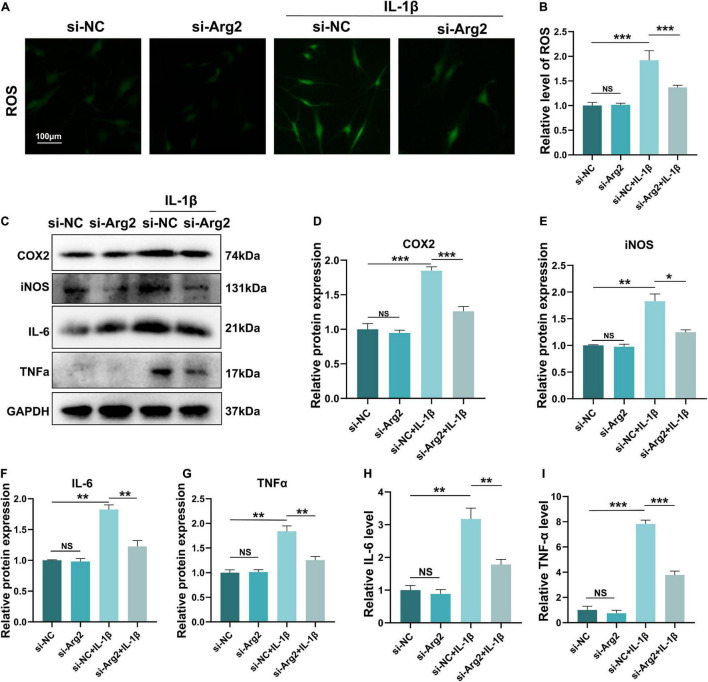
The effect of siRNA-ARG2 on oxidative stress and inflammatory response in NP cells. **(A,B)** The level of reactive oxygen species (ROS) induced by IL-1β was significantly reduced by ARG2 silencing (Scale bar: 100 μm). **(C–G)** The effect of downregulation of ARG2 on the levels of proinflammatory factors induced by IL-1β. **(H,I)** ELISA results detected the level of IL-6 and TNF-α. Data are represented as the mean ± SD. Two-group comparisons were analyzed by Student’s *t*-test. Significant differences between the treatment and control groups are indicated as *P < 0.05, **P < 0.01, and ***P < 0.001, *n* = 3.

Increased oxidative stress results in cellular apoptosis. ROS levels frequently represent intracellular oxidative conditions. To investigate the effects of ARG2 silencing on oxidative stress, we examined ROS levels in IL-1β-treated NPCs after treatment with siRNA-ARG2. IL-1β-induced ROS levels were significantly reduced by ARG2 silencing (Figures 5A,B), suggesting that the oxidative stress in NPCs was ameliorated *via* the downregulation of ARG2.

The inflammatory response that promotes both cellular senescence and apoptosis plays a vital role in the pathogenesis of IDD. In order to further examine the association between ARG2 and NPC inflammation, we examined the effect of ARG2 downregulation on IL-1β-induced proinflammatory factors. Our western blot data showed that IL-1β significantly enhanced the expression of COX2, iNOS, IL-6, and TNF-α. However, downregulation of ARG2 dramatically decreased COX2, iNOS, IL-6, and TNF-α levels compared with IL-1β treatment alone (Figures 5C–G). Consistent with these findings, our ELISA data also indicated that IL-1β treatment significantly increased IL-6 and TNF-α levels, while siRNA-ARG2 reversed these effects (Figures 5H,I). Taken together, our findings indicate that inhibition of ARG2 reduces inflammation in NPCs.

### The Role of Arginase 2 Overexpression in Nucleus Pulposus Cells

**FIGURE 6:**
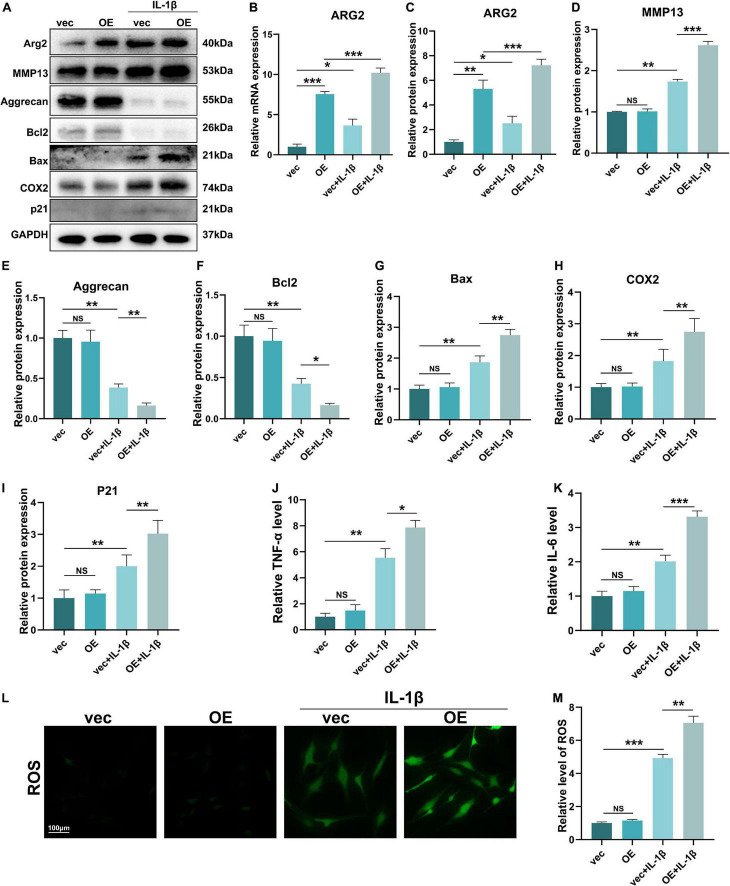
The role of ARG2 overexpression in NP cells. **(A)** The effect of ARG2 overexpression on the protein levels of ARG2, MMP13, aggrecan, Bcl2, Bax, COX2, and P21 in NPCs after the treatment of IL-1β. **(B)** The mRNA level of ARG2 in ARG2-overexpressed NPCs. **(C–I)** Quantitative analysis of western blotting results. **(J,K)** Supernatants of TNFα and IL-1β were tested with ELISA. **(L,M)** The ROS products was increased by ARG2 overexpression followed by treatment of IL-1β (Scale bar: 100 μm). Data are represented as the mean ± SD. Two-group comparisons were analyzed by Student’s *t*-test. Significant differences between the treatment and control groups are indicated as *P < 0.05, **P < 0.01, and ***P < 0.001, *n* = 3.

To further examine the effects of ARG2 upregulation on NPCs, we constructed an ARG2 overexpression plasmid (OE) and examined the effects of ARG2 on ECM metabolism, apoptosis and the inflammatory response. The efficiency of ARG2 overexpression was quantified by qPCR and western blotting. ARG2 expression levels were significantly higher in the OE group than in the vec group (Figures 6A–C). MMP13 and aggrecan are markers of ECM metabolism in NPCs. Overexpression of ARG2 led to a significant increase in IL-1β-induced MMP13 expression levels in NPCs (Figures 6A,D). Conversely, compared with the vec + IL-1β group, the level of aggrecan was markedly decreased in the OE + IL-1β group (Figures 6A,E). Compared with the vec + IL-1β group, overexpression of ARG2 in the OE + IL-1β group resulted in a significant decrease in the anti-apoptotic marker Bcl2, while the apoptotic marker Bax was markedly increased, suggesting that apoptosis was exacerbated in NPCs (Figures 6A,F,G). COX2 protein levels were also markedly higher in the OE + IL-1β group than the vec + IL-1β group (Figures 6A,H). The senescence-associated gene p21 was also found to be significantly upregulated in the OE + IL-1β group compared with the vec + IL-1β group (Figures 6A,I). To further elucidate the effect of ARG2 expression on the inflammatory response, TNFα and IL-1β levels in the cell culture supernatants were measured by ELISA. Both TNFα and IL-6 levels were significantly increased in IL-1β-treated cells overexpressing ARG2 (Figures 6J,K). ROS generation was also increased in the OE + IL-1β group (Figures 6L,M). Our findings indicate that overexpression of ARG2 increased ECM degradation, apoptosis, and inflammation in IL-1β-treated NPCs.

### Arginase 2 Has a Role in Interleukin 1β-Induced Oxidative Stress, the Inflammatory Response, and Apoptosis of Nucleus Pulposus Cells Through Activation of the NF−κB Pathway

**FIGURE 7:**
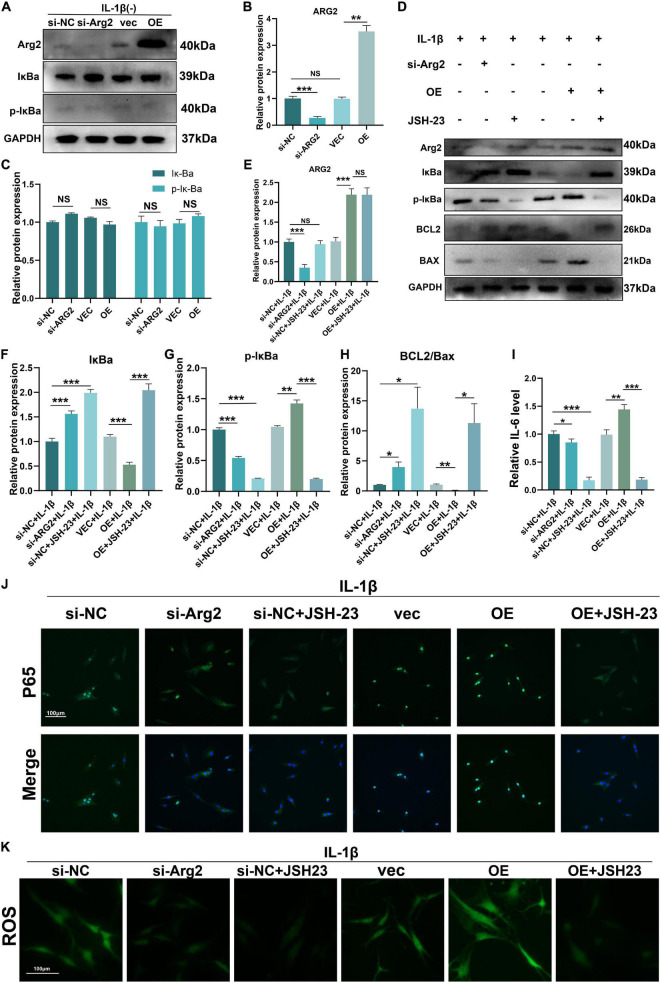
Role of ARG2 in IL-1β-induced oxidative stress, inflammatory response, and apoptosis of NPCs *via* the activation of NF–κB pathway. **(A–C)** The effect of ARG2 overexpression on NF-κB. **(D)** The levels of ARG2, NF-κB and apoptosis related proteins in the human NPCs were measured by western blotting. **(F–I)** Quantitative analysis of western blotting results. **(J)** The intracellular localization of p65 was detected by immunofluorescence (Scale bar: 100 μm). **(J,K)** The levels of IL-6 and TNFα were detected by ELISA. **(K)** The level of ROS was detected (Scale bar: 100 μm). Data are represented as the mean ± SD. Two-group comparisons were analyzed by Student’s *t*-test. Significant differences between the treatment and control groups are indicated as *P < 0.05, **P < 0.01, and ***P < 0.001, *n* = 3.

The NF-κB pathway plays a critical role in oxidative stress and apoptosis in NPCs (Kang et al., 2017; Zhu et al., 2019). Furthermore, some studies have indicated that ARG2 is involved in activation of the NF−κB pathway (Grandvaux et al., 2005). Thus, we sought to determine whether ARG2 regulates the NF−κB pathway in NPCs following treatment with IL-1β. Normally, NF-κB combines with IκBα, thereby inhibiting the nuclear translocation of NF-κB p65. Here, ARG2 protein levels were significantly elevated after overexpression of ARG2 (Figures 7A,B). In the absence of IL-1β, ARG2 did not affect the phosphorylation and degradation of IκBα (an endogenous NF-kB inhibitor) (Figures 7A,C). However, as shown in Figures 7D,F,G, in the presence of IL-1β, silencing ARG2 expression led to a decrease in phosphorylated IκBα, while ARG2 overexpression enhanced phosphorylation of IκBα (Figures 7D,F,G). Immunofluorescence staining was used to determine the intracellular localization of p65 (a major component of NF-κB) (Figure 7J). After IL-1β treatment, reduced p65 nuclear localization was observed in ARG2-silenced NPCs, whereas increased p65 translocation into the nucleus was found in ARG2-overexpressing NPCs relative to control NPCs. To further validate the involvement of NF-κB signaling in the ARG2-mediated inflammatory, oxidative stress, and cellular apoptosis responses, JSH-23 (a small molecule inhibitor targeting NF-κB pathway) was used. As shown in Figures 7D,E, JSH-23 treatment did not affect ARG2 protein expression (Figures 7D,E), but did result in a significant decrease in the amount of nuclear p65 (Figure 7J). Overexpression of ARG2 inhibited Bcl2 expression and promoted Bax expression (Figures 7D,H). However, inhibition of the NF-κB pathway with JSH-23 reversed the effect of ARG2 overexpression on Bcl2 and Bax levels (Figures 7D,H). IL-1β facilitates the release of inflammatory mediators. Using ELISA to detect levels of inflammatory factors, we found that IL-6 levels were significantly increased in ARG2-overexpressing cells treated with IL-1β (Figure 7I). However, inhibition of the phosphorylation of IκBα led to a significant reduction in IL-6 levels. We also found that JSH-23 significantly decreased ROS levels (Figure 7K). Taken together, our data suggest that ARG2 promoted IL-1β-induced oxidative stress, inflammatory response, and apoptosis through the NF-κB pathway.

## Discussion

The mechanisms underlying the development of IDD are complex. Pathological changes involve cellular senescence, apoptosis, inflammatory response, oxidative stress, and ECM degradation. Previously, ARG2 has been reported to regulate osteoarthritis pathogenesis by regulating the levels of matrix degrading enzymes in chondrocytes (Choi W. S. et al., 2019). Further studies have demonstrated that the inflammatory response is induced by upregulating ARG2 in the central nervous system (Choudry et al., 2018). However, the role of ARG2 in IDD remains unclear. Here, we found that downregulation of ARG2 alleviates apoptosis of NPCs, as well as promoting the release of proinflammatory cytokines including IL-6 and TNFα through activation of the NF-κB pathway, which accelerates ECM degradation and impairs NPC function.

Interleukin 1β plays a major role in the proinflammatory response and can promote the release of various proinflammatory mediators such as TNFα (Wang et al., 2020). Increased IL-1β levels have been reported in degenerative disks (Lin et al., 2019). IL-1β is strongly associated with the pathological development of IDD, including matrix degradation, inflammation, cellular senescence, apoptosis, and oxidative stress (Wang et al., 2020). Studies have indicated that various pathological factors such as diabetes, osteoarthritis, and IDD induce cellular senescence, apoptosis, inflammatory response, and oxidative stress *via* IL-1β (Al Ghamdi et al., 2015; Wang et al., 2018). In this study, ARG2 was upregulated by IL-1β treatment (Figure 2A), suggesting that ARG2 is involved in the IL-1β-induced phenotypic abnormalities of human NPCs.

Extracellular matrix catabolism is upregulated and anabolism is downregulated in the pathogenesis of IDD. A reduction in collagen and elevation in matrix-degrading enzymes occurs during IDD (Richardson et al., 2009). Here, downregulation of ARG2 not only enhanced type II collagen and aggrecan levels, but also inhibited the catabolism of ECM by downregulating matrix-degrading enzymes, suggesting that siRNA-ARG2 may have a protective effect on IDD (Figure 3). Previously, the rate of apoptosis in human NPCs obtained from IDD specimens was found to be increased by 53–73% (Huang et al., 2019). Apoptosis could be activated through the mitochondrial pathway by various stimuli (Zhao et al., 2006). In the present study, we compared apoptosis in degenerative NPCs to normal NPCs (Figure 4). We evaluated Bax, cleaved-caspase3, and Bcl2 levels to determine the role of ARG2 in the regulation of apoptosis. Our data demonstrated that Bcl2 was decreased but Bax and cleaved-caspase3 were increased in IL-1β-treated NPCs, and these effects were reversed with siRNA-ARG2 treatment. Cellular senescence is another crucial factor in the development of IDD. SA-β-gal, p21, and P16INK4a are reliable and classical markers for cellular senescence. Our data showed that downregulation of ARG2 could significantly reduce SA-β-gal, P16INK4a, and p21, suggesting that siRNA-ARG2 markedly inhibits IL-1β-induced senescence (Figure 4).

Oxidative stress, an activator of apoptosis (Hu et al., 2019), is an imbalanced status between products of oxidation and antioxidant defenses, which contributes to an increase in ROS. High levels of oxidation products have been reported in degenerated intervertebral disks (Che et al., 2020). Increased ROS levels result in destruction of the ECM of the intervertebral disk, thereby impairing the function of NPCs, which contribute directly to the development of IDD. In this study, our data indicated that downregulation of ARG2 led to inhibition of ROS, suggesting that inhibition of ARG2 could ameliorate the degeneration of intervertebral disks. In addition, a robust inflammatory response leads to marked elevation of inflammatory factors, including TNFα, IL-6, and IL-1β, which result in the inflammation-induced apoptosis of NPCs (Wu et al., 2020; Huang et al., 2021). It has been reported that the inflammatory response contributes to NPC death and degradation of the ECM (Wang et al., 2019). Here, we found that ROS and inflammation-related factors such as COX2, iNOS, IL-6, and TNFα were markedly elevated after IL-1β treatment. However, this trend was reversed by downregulation of ARG2, suggesting that ARG2 is closely associated with the regulation of ROS, inflammation, and apoptosis in NPCs.

To further determine the role of ARG2 in IDD, we transfected NPCs with an ARG2 OE. Western blot and real-time PCR studies showed that both ARG2 mRNA and protein levels were significantly increased in ARG2-overexpressing cells, indicating that our ARG2 overexpression system was successfully constructed. Moreover, overexpression of ARG2 resulted in increased protein expression of degeneration-associated factors such as MMP13 and aggrecan. In addition, markers of apoptosis, cellular senescence, the inflammatory response, and oxidative stress were also increased in ARG2-overexpressing NPCs. These results indicated that high levels of ARG2 impaired the normal function of NPCs and indirectly confirmed that ARG2 silencing is beneficial in the prevention of IDD.

NF-κB has been associated with the release of inflammatory cytokines involved in IDD (Yi et al., 2019) and plays a critical role in senescence, inflammation, and stress responses (Wu et al., 2018). Activation of NF-κB results in the upregulation of pro-inflammatory cytokines and matrix-degrading enzymes in IDD (Yi et al., 2019). Furthermore, inhibition of NF-κB could restrict the upregulation of MMPs and ADAMTS and degradation of collagen II and proteoglycan induced by IL-1β (Zhongyi et al., 2015). Based on previous studies, we examined whether ARG2 was associated with the NF-κB pathway in IDD. Our data showed that overexpression of ARG2 led to significant activation of NF-κB. In contrast, silencing of ARG2 with siRNA led to inhibition of NF-κB. Together, these results implicate ARG2 in the modulation of the NF-κB pathway.

However, it still remains unclear how ARG2 promotes IDD *via* NF-κB. In addition, the mechanism by which overexpression of ARG2 promotes NPC apoptosis and senescence needs further investigation. Our study was mainly conducted in human NPCs and tissues, so the effect of ARG2 on IDD *in vivo* cannot be fully explained. Future studies should be performed in ARG2 KO mice to address this issue.

In summary, our study shows that ARG2 is expressed in human NPCs and that its expression is increased in degenerated intervertebral disks. Inhibition of ARG2 *via* siRNA prevented apoptosis, inflammatory responses, oxidative stress, and senescence of human NPCs and ameliorated degradation of the ECM. In contrast, overexpression of ARG2 promoted apoptosis and inflammatory responses in human NPCs. Our findings provide a novel insight into potential therapeutic strategies for IDD. In particular, inhibition of ARG2 could be considered as a novel target for the prevention and treatment of IDD.

## Data Availability Statement

The raw data supporting the conclusions of this article will be made available by the authors, without undue reservation.

## Ethics Statement

The studies involving human participants were reviewed and approved by the Institutional Review Board of Changzheng Hospital of the Second Military Medical University. The patients/participants provided their written informed consent to participate in this study.

## Author Contributions

JGS and KQS designed the experiments. FDL, XFS, and BZ carried out the experiments. XFS, BZ, FL, CLJ, LH, XL, ZMS, and CY helped to collect the samples. KQS, JZ, JSX, YH, XMX, JCS, YW, and ZMS proofread the manuscript. FDL and FQK supervised the experiments, analyzed the results, and wrote the manuscript. All authors contributed to the article and approved the submitted version.

## Conflict of Interest

The authors declare that the research was conducted in the absence of any commercial or financial relationships that could be construed as a potential conflict of interest.

## Publisher’s Note

All claims expressed in this article are solely those of the authors and do not necessarily represent those of their affiliated organizations, or those of the publisher, the editors and the reviewers. Any product that may be evaluated in this article, or claim that may be made by its manufacturer, is not guaranteed or endorsed by the publisher.
